# 3D free-hand ultrasound to register anatomical landmarks at the pelvis and localize the hip joint center in lean and obese individuals

**DOI:** 10.1038/s41598-021-89763-7

**Published:** 2021-05-20

**Authors:** Brian Horsak, Caterine Schwab, Sebastian Durstberger, Alexandra Thajer, Susanne Greber-Platzer, Hans Kainz, Ilse Jonkers, Andreas Kranzl

**Affiliations:** 1 Institute of Health Sciences, St. Pölten University of Applied Sciences, St. Pölten, Austria; 2 Laboratory of Gait and Movement Analysis, Orthopedic Hospital Vienna-Speising, Vienna, Austria; 3 Department of Pediatrics and Adolescent Medicine, Medical University of Vienna, Vienna, Austria; 4 Department of Biomechanics, Kinesiology and Applied Computer Science, University of Vienna, Center for Sports Science and University Sports, Vienna, Austria; 5 Department of Movement Sciences, Human Movement Biomechanics Research Group, KU Leuven, Leuven, Belgium

**Keywords:** Orthopaedics, Medical research, Bone, Skeleton, Biomedical engineering

## Abstract

3D free-hand ultrasound (3DFUS) is becoming increasingly popular to assist clinical gait analysis because it is cost- and time-efficient and does not expose participants to radiation. The aim of this study was to evaluate its reliability in localizing the anterior superior iliac spine (ASIS) at the pelvis and the hip joint centers (HJC). Additionally, we evaluated its accuracy to get a rough estimation of the potential to use of 3DFUS to segment bony surface. This could offer potential to register medical images to motion capture data in future. To evaluate reliability, a test–retest study was conducted in 16 lean and 19 obese individuals. The locations of the ASIS were determined by manual marker placement (MMP), an instrumented pointer technique (IPT), and with 3DFUS. The HJC location was also determined with 3DFUS. To quantify reliability, intraclass correlation coefficients (ICCs), the standard error of measurement (SEm), among other statistical parameters, were calculated for the identified locations between the test and retest. To assess accuracy, the surface of a human plastic pelvic phantom was segmented with 3DFUS in a distilled water bath in 27 trials and compared to a 3D laser scan of the pelvis. Regarding reliability, the MMP, but especially the IPT showed high reliability in lean (SEm: 2–3 mm) and reduced reliability in obese individuals (SEm: 6–15 mm). Compared to MMP and IPT, 3DFUS presented lower reliability in the lean group (SEm: 2–4 mm vs. 2–8 mm, respectively) but slightly better values in the obese group (SEm: 7–11 mm vs. 6–16 mm, respectively). Correlations between test–retest reliability and torso body fat mass (% of body mass) indicated a moderate to strong relationship for MMP and IPT but only a weak correlation for the 3DFUS approach. The water-bath experiments indicated an acceptable level of 3.5 (1.7) mm of accuracy for 3DFUS in segmenting bone surface. Despite some difficulties with single trials, our data give further rise to the idea that 3DFUS could serve as a promising tool in future to inform marker placement and hip joint center location, especially in groups with higher amount of body fat.

## Introduction

In clinical gait analysis, biomechanical models assume that markers are placed above bony landmarks. The combination of a minimum of three markers are used to define body segment coordinate systems. The relative movement of the skin to the bone and wobbling mass, so-called soft tissue artifacts (STA), as well as inaccurate and unreliable marker placement can strongly affect the accuracy of 3D gait analysis^1–4^. These errors can have clinically relevant effects on estimated HJC locations and potentially introduce errors in tracking body segments during locomotion^5–9^. An alternative to manual marker placement (MMP) is the calibrated anatomical systems technique^10^, referred to in this study as the “instrumented pointer technique (IPT)”. It consists of a pointer with a minimum of two markers attached to it and allows to digitize the tip of the pointer as a 3D coordinate in a reference coordinate system. In clinical practice it is primarily used to define anatomical landmarks, which are prone to STA. For example, the IPT is used to define the anterior superior iliac spine (ASIS) as virtual markers within a cluster-based reference coordinate system placed at the sacrum^11^. A big advantage of these virtual ASIS markers is that they are only prone to the STA introduced by the sacrum cluster which is expected to be lower compared to STA at the abdominal region. Thus, more reliable results are expected. Tawy and Rowe^12^ have demonstrated that the IPT is a reliable method, but they only used a box as a measurement phantom to evaluate reliability. However, clinical practice may involve determining and palpating anatomical structures in individuals with a high amount of subcutaneous fat. This makes the identification of the underlying anatomical structure challenging and presumably both, MMP and IPT, more unreliable^2,3^.

Three dimensional free-hand ultrasound (3DFUS) is a combination of a conventional ultrasound probe with a 3D motion capture system. It enables the transformation of 2D pixel coordinates of an ultrasound image to the 3D coordinate system of the motion capture system^13^. 3DFUS can be used to measure geometrical aspects of the human skeletal system and identify locations of anatomical landmarks without being affected by soft tissue. This information then can be used to personalize biomechanical models. Compared to volumetric medical imaging such as computed tomography (CT) or magnetic resonance imaging (MRI), 3DFUS offers a potentially cost- and time-efficient option in measuring in-vivo aspects of the human skeletal system without exposure to radiation. These factors make 3DFUS an attractive possibility in clinical and research settings for gait analysis and may be the reason for the increased popularity in recent years. 3DFUS has proved to be an applicable approach in 3D gait analysis to localize the hip joint centers (HJC)^14,15^, identify the medio-lateral axis of the femur^16^, measure femoral torsion^17^, and was used to create subject-specific models by measuring bone geometry and muscle-tendon architecture^18^. As already demonstrated by Passmore et al.^18^ and Jia et al.^19^, 3DFUS can also be used to identify bone geometry of the femur condyles or the trochanter major. 3DFUS is also being used in other research domains, i.e., to visualize anatomical structures in 3D, measure quantitative 3D attributes, and for fusion with other 3D image modalities. A review by Mozaffari and Lee^20^ provides a general overview. Recently, it was outlined by Kubicek et al.^21^ that in particular in the domain of computer assisted orthopedic surgery 3DFUS is being successfully used to identify anatomical landmarks, more particular to measure pelvic tilt or to track anatomical planes to inform surgeons. 3DFUS can reliably identify pelvic tilt during standing, sitting and in supine position^22,23^ with an accuracy in the range of approximately 0$^{\circ }$–4$^{\circ }$^24,25^ and with good to excellent reliability^25,26^. Research also demonstrated that 3DFUS-based pelvic tilt tracking is less affected by higher amounts of soft tissue than traditional pointer-based imaging modalities^26,27^. These results are encouraging and leverage the relevance to further investigate the potential of 3DFUS to support clinical and research practice in the 3D gait analysis community. Yet, the currently available 3DFUS related research in clinical 3D gait analysis is mostly limited to normal weighted study groups. However, increased subcutaneous fat makes the identification of anatomical structures more challenging. Research on the application of 3DFUS in individuals with increased body mass is scarce, has a limited number of participants, or was used to measure pelvic tilt only for the purpose of computer assisted orthopedic surgery^24^ and did not compare to skin-marker-based measurements. In clinical gait analysis, the location of the ASIS landmarks is highly important as these locations are required to define the pelvic anatomical coordinate system. Furthermore, ASIS locations are used to calculate pelvic width, which is subsequently used to estimate the HJC location, i.e., via regression equations^28^. Currently available research on 3DFUS does not report any details on reliability in identifying ASIS locations.

Therefore, this study adds to the 3DFUS-related research field by evaluating the reliability of a 3DFUS-based approach to identify the ASIS locations and the HJC for the purpose of clinical 3D gait analysis. In contrast to earlier research, we conducted our 3DFUS experiments in a large number of lean and obese participants with the purpose to investigate the utility of 3DFUS in the context of applying external markers and in identifying the HJC location for clinical gait analysis. The reliability in identifying the ASIS landmarks was also compared to MMP and the IPT. We hypothesized that placing markers manually and using the IPT, both will be either similar or even more reliable in the lean study sample compared to 3DFUS. However, we also expected that errors will increase to a lesser extent for the 3DFUS-based approach in obese individuals and therefore placing it as the superior method in this sample. The reason to hypothesize that 3DFUS will not be superior in lean participants is associated with the difficulties in using the probe on edgy bony landmarks with little body fat covering them. Additionally, we also conducted simple water-bath experiments with a plastic measurement phantom of a pelvis to get a rough estimation of the potential usability of 3DFUS in segmenting entire surface areas of bony landmarks. As highlighted by the work of Gasparutto et al.^29^, this might in future allow to register medical images such as MRI or CT scans to motion capture data.

## Materials and methods

### Material

**Figure 1:**
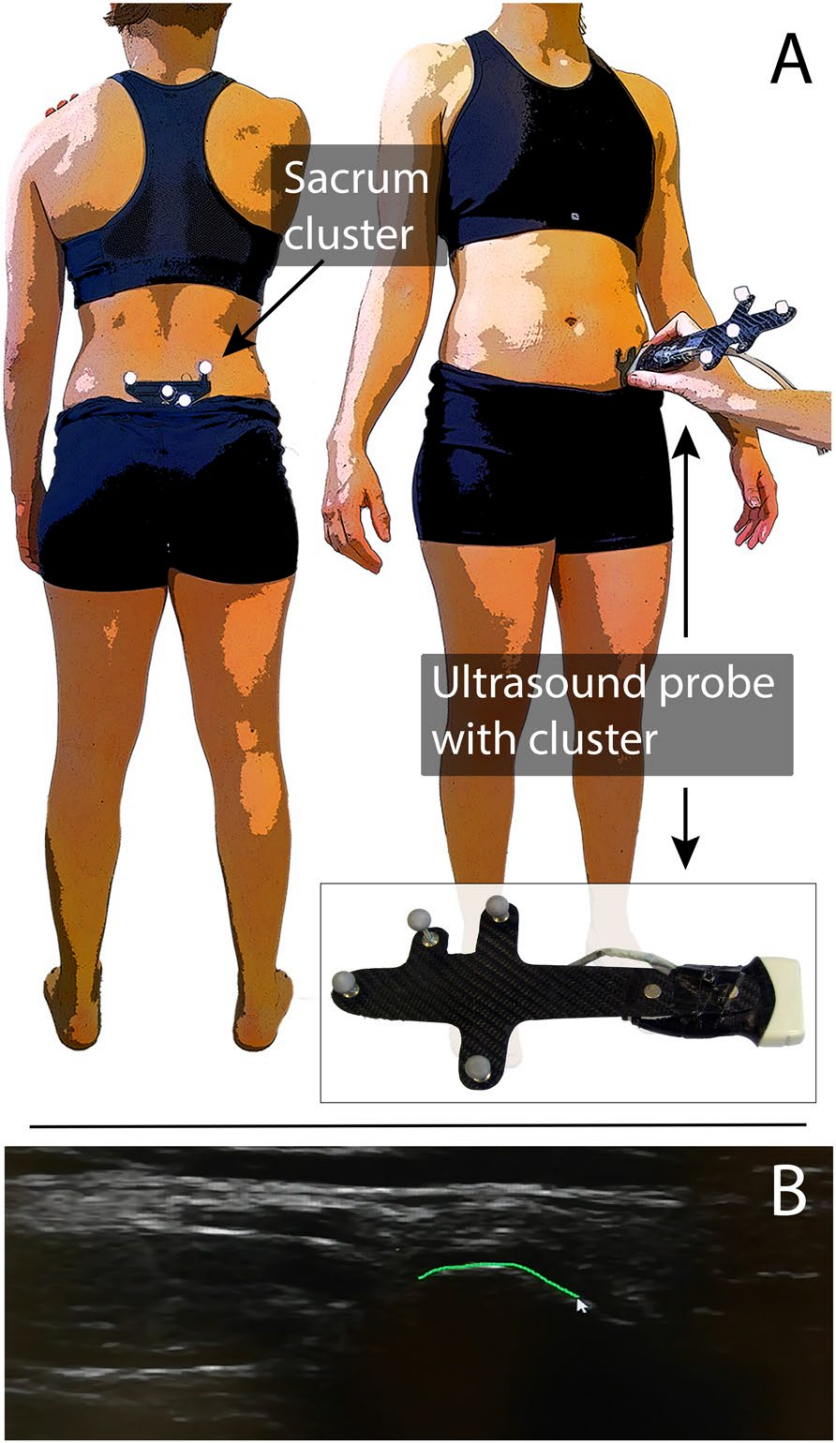
(**A**) The ultrasound probe was equipped with a marker-cluster to transform the image coordinates to the motion-capture coordinate system. A marker-cluster placed at the sacrum of the participants served as the local reference system. (**B**) This image illustrates how the pixel coordinates of the bone surface, in this case of the anterior superior iliac spine (ASIS), in each ultrasound image were manually digitized. Note that for the identification of the ASIS location the most anterior point was selected manually.

The utilized ultrasound system was a Telemed ClarUS EXT (http://www.telemedultrasound.com/) unit synchronized to a 10-camera Vicon TX10 system (Oxford, UK) operating at 150 Hz. We have used a linear probe (L12-5L40N-4) with a frequency range of 5–12 MHz and field of view of 39 mm. The ultrasound probe was equipped with a rigid 4-marker-cluster (see Fig. 1). After initial calibration^30^, it allowed to transform the pixels of the ultrasound images to 3D coordinates of the motion capture system. All marker data were filtered using the Woltring Generalized Cross-Variance filter with a smoothing level of 20, which is embedded in Vicon Nexus 2.8.

**Table 1:** Overview of the study sample’s characteristics.

	Lean group	Obese group
N	16	19
Sex (N)	m = 8	m = 12
	f = 8	f = 7
Age (years; SD, range)	13.8 (2.8, 10–18)	13.2 (2.6, 10–18)
Body mass (kg)	47.0 (12.5)	85.5 (18.9)
Height (m)	1.59 (0.1)	1.61 (0.1)
BMI (kg/$\hbox {m}^2$)	18.3 (2.7)	32.7 (5.3)
Fat mass (%)	15.0 (8.5)	37.9 (6.3)
Fat free mass (%)	85.0 (8.5)	62.1 (6.3)
Fat mass torso (%)	14.9 (10.0)	43.3 (7.9)
Fat free mass torso (%)	41.5 (7.2)	30.5 (4.5)

Values are reported as mean (SD), unless otherwise stated.

### Participants

To evaluate the test–retest reliability, we recruited a convenience sample of 16 lean and 19 obese children and adolescents, see Table 1. All obese participants were right at or above the 95th percentile of the central Europe specific sex- and age-based BMI and considered as obese^31^. As initial tests did not indicate any statistically significant differences, data from the left and right body side were treated as independent observations, resulting in 32 and 38 legs for the lean and obese group, respectively. This study was approved by the local ethics committee of the Medical University of Vienna (2060/2017) and was performed in accordance with the relevant guidelines and regulations. All participants, and when necessary their legal representatives (< 18 years of age), were informed prior to the study and gave written informed consent.

### Reliability

Participants were assigned to a single test day to the laboratory. During that day, two sessions which were at least one hour apart, were recorded. During the first session, segmental full-body composition was measured with a bioelectrical impedance analysis (BIA) device (Biacorpus RX 4000, Medical Healthcare GmbH, Germany). For this purpose participants lay horizontally, with legs and arms slightly apart from the body, for five minutes without any movements. Eight adhesive electrodes, two on each limb, were attached and allowed to measure total and torso fat mass and fat free mass in percentage of body mass (%), see Table 1. During the test and retest session, the ASIS locations were determined by one assessor with three methods: (1) by manual palpation and marker placement (MMP), (2) by using the IPT, and (3) by using the 3DFUS approach. In addition, 3DFUS was also used to determine the HJC position (3DFUS-HJC) by sweeping with the ultrasound probe perpendicular to the skin at the inguinal region horizontally and vertically. For this purpose participants stood in external hip rotation to expose the anterior surface of the femoral heads^15^. During data analysis of the 3DFUS-HJC trials we had to remove 11 trials (legs) from the obese and two trials (legs) from the lean group due to problems with image quality and a technical problem, respectively. In the obese cases the HJC were located too deep. This prevented us to acquire images with sufficient quality. A minimum of approximately 10 and a maximum of 50 ultrasound image frames were used to digitize the curvature of the femoral heads. A M-estimator Sample Consensus (MSAC) algorithm^32^ was then used to fit a sphere into these data to determine the location of the femoral heads (HJC). ASIS were defined by selecting the most anterior structure manually in the ultrasound images. The assessor had several years of experience in 3D gait analysis and approximately four months of experience with the 3DFUS system. A rigid marker-cluster attached to the sacrum during the test and which stayed in place for the retest, served as a common reference system, see Fig. 1. To quantify reliability, Intraclass Correlation Coefficients (ICC), the Standard Error of Measurement (SEm), as well as the Euclidean distances and the differences between test and retest of the identified coordinates were calculated. SEm was derived from a two-way repeated ANOVA^33,34^. Note that the SEm should not be confused with the Standard Error of the Mean (SEM). ICCs were calculated based on a single-measure, absolute-agreement, and two-way mixed effects model ($\hbox {ICC}_{2,1}$). ICC values of less 0.5 were rated as poor, values between 0.5 and 0.75 as moderate, values between 0.75 and 0.9 as good, and values greater than 0.90 as excellent reliability. For more details to ICCs the reader might refer to^33,35^.

To statistically evaluate if test–retest errors in terms of the Euclidean distances differed between the methods to localize the ASIS (MMP, IPT, and 3DFUS) a Friedman test was conducted for the lean and the obese group separately. A Shapiro–Wilk test indicated violation of normality for several variables which was the reason to use non-parametric tests. In case of a significant result, additional pairwise Mann-Whitney tests were performed. To avoid multiple testing and, thus, high family-wise error rates, tests were only performed for the Euclidean distances. However, to reduce type II errors, post hoc tests were not Bonferroni-corrected. Alpha level was set to 0.05.

For a more intuitive interpretation, the data were expressed in an local pelvis coordinate system based on the sacrum-cluster. Pearson product–moment correlations were run to determine the relationship between the test–retest Euclidean distances for all methods (MMP, IPT, 3DFUS, and 3DFUS-HJC) and fat mass of the torso (FMT).

### Accuracy

To evaluate the accuracy of the 3DFUS system in reconstructing bone surface, 27 recordings of a human plastic pelvis measurement phantom placed in a distilled water bath were recorded. Areas near the ASIS, posterior iliac spines, and the HJC of the measurement phantom’s surface were recorded by one single assessor for approximately three trials a day, on nine different days. During these scans we did not put specific focus on the ASIS and HJC alone, rather than scanning also surrounding areas near the ASIS and HJC of the phantom. The bone surface in the 3DFUS images were manually digitized in every 10th frame of the ultrasound recording by using a digitizing pen and a Microsoft Surface Pro 3 tablet (Microsoft Corporation, USA). The velocity of sound in water was calculated by the formula of Lubbers and Graaff^36^. To quantify accuracy, a 3D laser scan (Atos Core 300, GOM GmbH, Germany, resolution: 0.12 mm) of the pelvis phantom served as the ground truth (see Fig. 4). A 3D-printed marker cluster glued to the pelvis was used as a common reference coordinate system. We determined the accuracy of the 3DFUS technique by calculating the minimal Euclidean distances between the 3DFUS point-clouds and the 3D laser scan point-cloud for each trial and summarized them by descriptive statistics. Outliers of the minimal Euclidean Distances were defined as points which are more than 1.5 times of the interquartile range away from the 25th and 75th quartile. Within all 27 recordings, only 6.2% of the points were classified as outliers.

## Results

**Figure 2:**
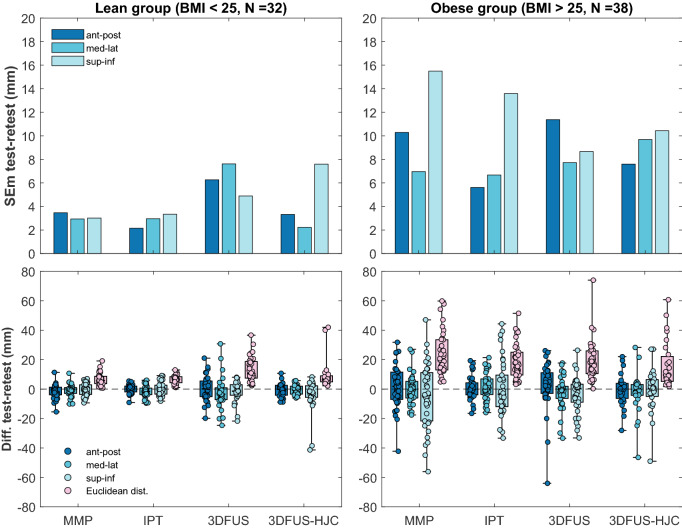
The upper plots show the Standard Error of Measurement (SEm) as an indicator for test–retest reliability for the anterior superior iliac spine (ASIS) location in all three anatomical planes. The lower plots show the distances between test and retest of the ASIS location in all three anatomical planes as well as the Euclidean distances between test and retest. Plots show a boxplot (with the median) and the raw data. ASIS were determined by manual marker placement (MMP), an instrumented pointer technique (IPT), and by the 3D free-hand ultrasound approach (3DFUS). The hip joint center (HJC) was localized by 3DFUS. Analyses were performed within a lean (left plots) and an obese study sample (right plots). Note that 11 trials (legs) from the obese and two trials (legs) from the lean group had to be removed for 3DFUS-HJC due to problems with image quality and a technical problem, respectively.

**Table 2:** The intraclass correlation coefficients ($\hbox {ICC}_{2,1}$) and their 95% confidence intervals (CI) with lower and upper bounds (LB, UB) for manual marker placement (MMP), the instrumented pointer technique (IPT), and 3D free-hand ultrasound (3DFUS) to identify the anterior superior iliac spine (ASIS) as well the 3DFUS based hip joint center (HJC) estimation.

	Lean group		Obese group	
	$\hbox {ICC}_{2,1}$	95% CI (LB–UB)	$\hbox {ICC}_{2,1}$	95% CI (LB–UB)
**MMP**				
Ant-post	0.97	(0.93–0.98)	**0**.**84**	(0.71–0.91)
Med-lat	1.00	(1.00–1.00)	1.00	(1.00–1.00)
Sup-inf	0.98	(0.96–0.99)	**0**.**76**	(0.59–0.87)
**IPT**				
Ant-post	0.98	(0.97–0.99)	0.94	(0.90–0.97)
Med-lat	1.00	(1.00–1.00)	1.00	(0.99–1.00)
Sup-inf	0.97	(0.94–0.99)	**0**.**77**	(0.60–0.87)
**3DFUS**				
Ant-post	0.90	(0.81–0.95)	**0**.**82**	(0.68–0.90)
Med-lat	0.99	(0.99–1.00)	1.00	(0.99–1.00)
Sup-inf	0.93	(0.87–0.97)	0.92	(0.85–0.96)
**3DFUS-HJC**				
Ant-post	0.97	(0.94–0.99)	0.91	(0.80–0.96)
Med-lat	1.00	(1.00–1.00)	0.99	(0.97–0.99)
Sup-inf	**0**.**77**	(0.56–0.89)	**0**.**89**	(0.78–0.95)
**Total**				
Mean	0.96	(0.91–0.98)	0.90	(0.82–0.95)
Min	0.77	(0.56–0.89)	0.76	(0.59–0.87)
Max	1.00	(1.00–1.00)	1.00	(1.00–1.00)

ICCs were calculated between test and retest. Values lower than 0.9 were marked bold. Note that values are rounded to two decimal places which in cases of high ICCs renders values of 1.00.

**Figure 3:**
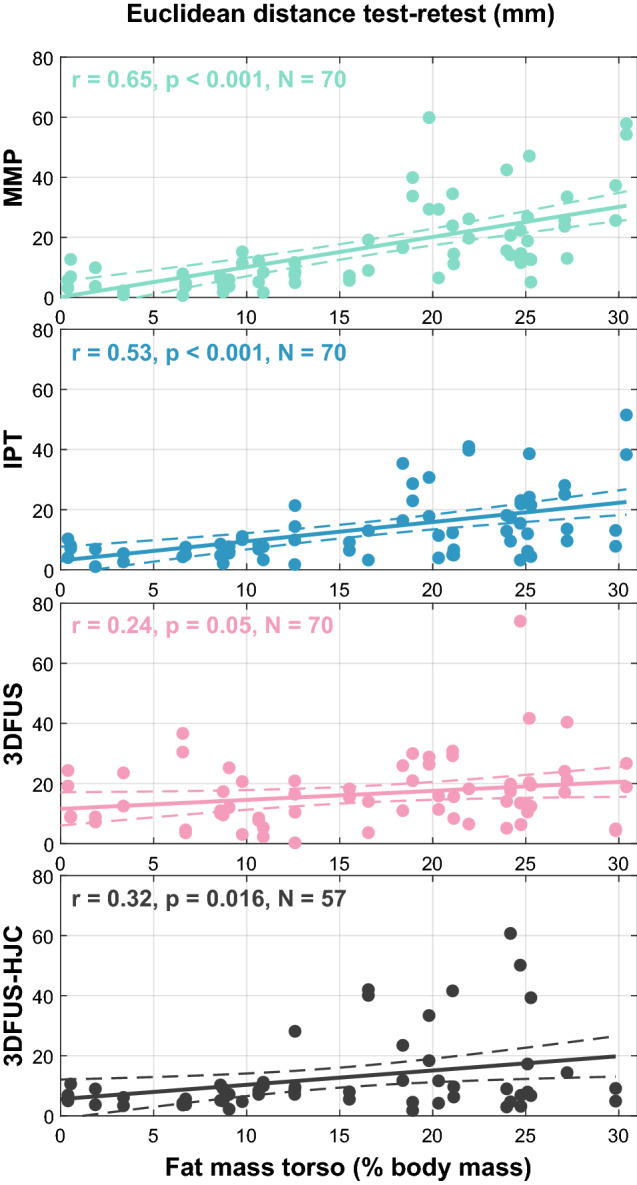
Euclidean distances of the anterior superior iliac spine (ASIS) and the hip joint center (HJC) location between the test and the retest plotted against the fat mass of the torso in % body mass. The first three plots show the methods, manual marker placement (MMP), instrumented pointer technique (IPT), and 3D free-hand ultrasound (3DFUS) to determine the ASIS positions. The fourth plot shows the 3DFUS-based HJC location. The solid line represents the line of best fit. The dashed lines show the 95% confidence intervals. Pearson’s r, the p values, and the number of included data points are shown for each plot. Note that 11 trials (legs) from the obese and two trials (legs) from the lean group had to be removed for 3DFUS-HJC due to problems with image quality and a technical problem, respectively.

ICCs values presented excellent values (> 0.93) for all methods in the lean group except for the superior–inferior direction of 3DFUS-HJC estimation which showed good agreement (0.77). ICCs values in the obese group were in general lower and ranged between 0.76 and 0.99 indicating good to excellent agreement. Details can be found in Table 2.

Results of the SEm and Euclidean distances for the lean and obese group and all methods are shown in Fig. 2. Statistical evaluation of the Euclidean distances indicated significant differences in test–retest error for locating the ASIS depending on which method was used in the lean group, $\chi {^2}$ (3) = 17.687, *p* < 0.001, but significance was slightly missed for the obese group, $\chi {^2}$ (3) = 4.895, *p* = 0.087. In the lean group the median (IQR) Euclidean distances for MMP, IPT, and 3DUS were 6 mm (3–9), 6 mm (4–8) and 10 mm (7–19), respectively. Post hoc tests indicated no significant difference between the MMP and IPT ($Z = -$ 0.374, *p* = 0.708). However, there was a statistically significant difference between MMP and 3DUS ($Z = -$ 3.403, *p* = 0.001) and between IPT and 3DFUS (*Z* = − 3.740, *p* < 0.001). In the obese group the median (IQR) Euclidean distances presented higher values for MMP and lower ones for IPT and 3DUS with 22 mm (13–34), 17 mm (10–26) and 17 mm (11–26), respectively. Regarding the SEm, in the obese group 3DFUS showed a lower SEm (9 mm) compared to MMP and IPT ($\sim$ 15 mm) in identifying the superior–inferior position of the ASIS. The medio-lateral location was comparable for all three methods ($\sim$7 mm). In anterior–posterior direction the IPT (6 mm) was superior to MMP (10 mm) and 3DFUS (11 mm). In addition, Table 3 gives an overview of the variation (standard deviation, SD) of the differences of the ASIS locations between test and retest for all methods and both study groups. These variations indicate that the 3DFUS approach was less affected by body fat in the obese group compared to the lean group as results presented a smaller change of variation (lean vs. obese) for the 3DFUS approach, compared to MMP and IPT. This is easiest seen by the Euclidean distances for each method in the third and fourth column of Table 3. Variation increased from the lean to the obese group 334% (+ 10 mm) and 416% (+ 9 mm) for MMP and IPT, respectively, but only 159% (+ 5 mm) for the 3DFUS based identification of the ASIS location. 3DFUS-HJC demonstrated an increase in variation of 174% (+ 6.6 mm) from the lean to the obese group. Pearson product-moment correlation indicated a moderate to strong, statistically significant, and positive correlation between the Euclidean distances and FMT for MMP (r = 0.65, p < 0.001) and IPT (r = 0.53, p < 0.001) and a weak positive correlation for 3DFUS (r = 0.24, p = 0.05) and the 3DFUS-HJC (r = 0.32, p = 0.016). See Fig. 3 for details.

**Figure 4:**
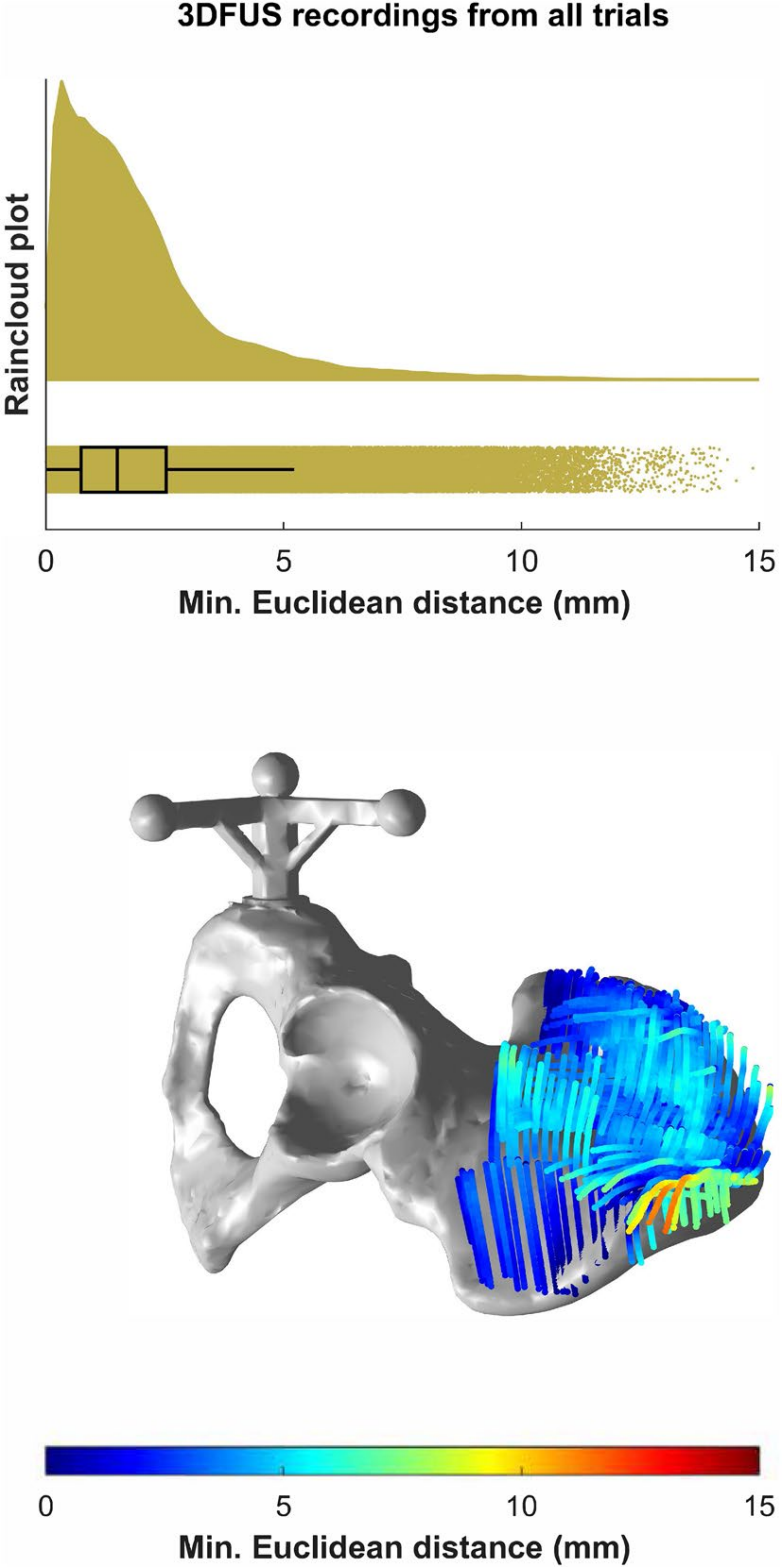
The upper plot shows a rain cloud plot of the minimum Euclidean distances between all 27 3DFUS bone segmentation trials and the laser scan of the measurement phantom (ground truth). Rain cloud plots combine an illustration of data distribution (probability density function), jittered raw data, and a boxplot (with the median)^37^. The lower plot shows a heatmap of the Euclidean distances between the measurement phantom and one single 3DFUS bone segmentation trial. Red colors show greater differences and blue colors smaller ones. The attached cluster of three markers served as a common reference system between the laser scan and the 3DFUS data.

The water-bath experiments indicated an acceptable level of accuracy of the 3DFUS technique in segmenting the surface of the pelvis phantom across all 27 trials. The mean (SD) of the minimum Euclidean distances between the laser scan model and all 3DFUS bone segmentation trials was 3.5 (1.7) mm with a range of 0–11.9 mm. Fig. 4 shows the point-cloud of the laser scan (ground truth) with an overlay of the segmented bone surface data of one 3DFUS trial and a rain-cloud boxplot^37^ for the minimum Euclidean distance results across all trials. The supplementary material 1 includes detailed plots for each trial separately.

**Table 3:** Overview of the variation (standard deviation, mm) of the anterior superior iliac spine (ASIS) and hip joint center (HJC) locations between test and retest (first two columns) as well as the absolute difference (mm) and relative difference (in% of the lean group) between both groups.

Variation	Lean group (mm)	Obese group (mm)	Absolute Diff. (mm) (|Obese$| -|$Lean|)	Relative Diff. (in % of the lean group)
**MMP**				
Ant-post	4.8	14.4	9.5	297
Med-lat	4.1	9.7	5.6	238
Sup-inf	4.2	21.6	17.4	516
Eucl. dist.	4.2	14.2	9.9	334
**IPT**				
Ant-post	3.0	7.8	4.8	261
Med-lat	4.1	9.3	5.2	226
Sup-inf	4.6	19.0	14.3	408
Eucl. dist.	2.9	12.0	9.1	416
**3DFUS**				
Ant-post	8.7	15.9	7.1	182
Med-lat	10.6	10.8	0.2	102
Sup-inf	6.8	12.1	5.3	178
Eucl. dist.	8.3	13.2	4.9	159
**3DFUS-HJC**				
Ant-post	4.6	10.5	5.9	228
Med-lat	3.1	13.4	10.3	435
Sup-inf	10.6*	14.5	3.9	137
Eucl. dist.	8.9*	15.5	6.6	174

*Note that there are two outliers (from one person) present, which contribute to the high values (see Fig. 2). *MMP* manual marker placement, *IPT* instrumented pointer technique *3DFUS* 3D free-hand ultrasound, *HJC* hip joint center.

## Discussion

This study evaluated the reliability of an 3DFUS-based approach in identifying anatomical landmarks at the pelvis and the location of the HJC. In addition, we also conducted simple water-bath experiments with a plastic measurement phantom of a pelvis to get a rough estimation of the potential usability of 3DFUS in reconstructing bony surface. For evaluating the reliability, the position of the ASIS were determined by manual marker placement (MMP), an instrumented pointer technique (IPT), and by a 3D free-hand ultrasound (3DFUS) technique. Test–retest experiments were carried out in a lean and an obese study sample. We hypothesized that MMP and the IPT will both be either similar or more reliable in the lean study sample compared to the 3DFUS approach. In addition, we expected that errors will increase to a lesser extent for the 3DFUS based approach in obese individuals and therefore placing it as the superior method in this sample. The obtained data support this hypothesis only partly.

In the lean study sample all methods demonstrated good to excellent reliability, indicated by the ICCs and the SEm. The latter indicated error-margins of less than 8 mm. As expected, the MMP and the IPT as well as the 3DFUS-based HJC localization were highly reliable in the lean group (see Fig. 2). Only the 3DFUS-based estimation of the ASIS presented slightly higher SEm errors in the lean group, but was also in an acceptable range (< 8 mm). This is in line with Peters et al.^14^ who reported a range of approximately 4 mm for their 3DFUS-based HJC reliability study. Studies originating from the field of computer assisted orthopedic surgery, where 3DFUS is used to identify pelvic tilt in various positions, also demonstrated that 3DFUS can be a reliable tool^24–27^. In example, Dardenne et al.^24^ have recently evaluated the intra- as well as inter-observer reliability of an ultrasound-based device to measure patient-specific pelvic tilt in different daily positions. They reported average ICC values of greater than 0.9, except for standing where a slightly lower ICC of 0.7 was found. Similar high reliability indices were found by Marques et al.^25^. They reported ICC values of greater than 0.85 and SEm values of approximately less than 4$^{\circ }$ to identify pelvic tilt in a normal weighted cohort of participants. Our results also compare well to Kainz et al.^28^ who have reported an inter-trial average difference of below 5 mm with the Harrington regression equation and below 20 mm for the geometric fit method to determine the HJC location. This shows that 3DFUS is able to reach the currently available standards in the identification of the HJC position in lean individuals. In an earlier study we showed that different non-invasive HJC estimation methods localized the HJC position with up to an alarming 6 cm of difference in an obese population^2^. These errors were primarily due to problems in identifying anatomical landmarks and STA. However, the higher variability and the presence of outliers in the 3DFUS-based estimates in the test–retest differences indicate a lower robustness of 3DFUS (see Fig. 2) compared to the manual techniques in identifying the ASIS locations and the HJCs. This observation is also in line with Peters et al.^14^. They used 3DFUS in a similar study with 53 children out of which seven participants had to be removed due to poor image quality.

As expected, the reliability error-margins, in terms of SEm and test–retest differences, increased throughout all methods in the obese group. Interestingly, the identification of the superior–inferior position of the ASIS showed the lowest reliability for MMP and IPT in the obese group (see Fig. 2). This is not completely surprising. It is very hard to identify the ASIS location in overweight individuals, but especially the identification of the superior–inferior position is difficult when one has hardly any reference points which could guide manual palpation. This is the case in obese individuals. Thus, this takes effect in all methods based on manual identification of anatomical landmarks (MMP and IPT). Even though, this was also seen in the 3DFUS-based approach, the SEm increased to a lesser extent from the lean to the obese group and values were even slightly lower ($\sim$ 5 mm) in the obese group compared to MMP and the IPT. This is partially in line with earlier research from the computer assisted orthopedic surgery domain, where researchers were able to show that 3DFUS-based navigation is almost not affected by increased soft tissue^24^ or at least superior to the traditional pointer-technique^27^. However, in comparison to that research we observed in general slightly lower reliability values for the obese group. One reason could be that we conducted all experiments during standing. Dardenne et al.^24^ have already demonstrated that reliability is lower when 3DFUS is performed during standing in comparison to a supine or sitting position. The standing position, in combination with the high amount of soft tissue in our study might have contributed to our lower results. However, as the motion capture system needs to see the sacrum cluster during scanning, a supine position—as adopted for computer assisted orthopedic surgery applications—is not feasible. Solving that issue in future would allow to increase reliability of 3DFUS in obese individuals for the purpose of ASIS and HJC localization in 3D gait analysis. The variability of the test–retest differences was also slightly lower for the 3DFUS-based methods compared to MMP and the IPT (see Table 3). Thus, at this point our data at least point toward the direction that 3DFUS could be a superior method in locating the ASIS compared to MMP and IPT, especially in obese individuals. On the other hand statistical analysis of the Euclidean distances did not render any significant differences between MMP, IPT, and 3DFUS in locating the ASIS. Yet, SEm might be a more sensitive estimate of reliability compared to the test–retest Euclidean distances, as it divides the test–retest variance into its components and thereby providing only error-variance as a measure of reliability. In addition, our ultrasound images were recorded and digitized by hand from a moderately experienced biomedical-engineer. Glor et al.^38^ already showed that the quality of ultrasound images depends on the experience of the assessor in performing recordings. Therefore, our 3DFUS approach might perform even better when the assessor is highly trained in acquiring and digitizing ultrasound images.

It is obvious that the reliability of all techniques was partly affected by FMT. This is clearly seen in the correlations between reliability (in terms of test–retest Euclidean distances) and the FMT in Fig. 3. All methods showed a positive correlation between test–retest errors and FMT. However, both 3DFUS-based approaches only showed a weak correlation whereas MMP and the IPT showed a moderate to strong correlation. This might indicate that the 3DFUS technique is less susceptible to errors introduced by body composition parameters such as body fat. However, it needs to be kept in mind that there might be additional factors at play which we are not aware of at the moment. For example, the force applied to the probe by the assessor might also influence the results. This was not controlled in this study. In addition, our experience showed us that obese individuals present a wide range of soft-tissue quality from high amount of visceral fat (located in the abdomen) to high amount of subcutaneous fat (located below the skin), and combinations. This resulted in different soft tissue properties from being very tight to very soft and could in turn also have modulated our results. However, we did not specifically measure this. Nonetheless, MMP and IPT outperformed the 3DFUS-based approach in identifying the ASIS location in the lean group. In our experience it is difficult to record ultrasound images especially above the ASIS, when subcutaneous fat is hardly present. It is then very difficult to rest the probe on the skin because the bone protrudes and does not offer a good base of support for the probe. It gets easier when there is at least a bit of soft tissue between the probe and the anatomical structure, e.g. when scanning the HJC in the lean group. This could be the reason why the variability of the 3DFUS-based approach in identifying the ASIS in the lean group is higher than of the 3DFUS-HJC, see Fig. 2. The use of ultrasound gel pads to give the ultrasound probe a better base of support during scanning might help to reduce this problem in future. Based on our obtained results, 3DFUS-based approaches seem to offer promising alternatives to localize anatomical structures in groups where high amount of subcutaneous fat is present, and where the assessor has difficulties in identifying these by palpation. In lean individuals, however, 3DFUS-based approaches might only be of advantage to identify the position of the HJC but don’t offer great advantages in identifying protruding anatomical structures such as the ASIS.

To investigate the accuracy of our 3DFUS-based approach in segmenting bone surface we have conducted extensive water-bath experiments with a plastic pelvis measurement phantom. A 3D laser scan of this phantom served as the ground truth. The results indicated an acceptable level of accuracy ($\sim$3.5 mm, see Fig. 4) throughout all recorded trials of the 3DFUS in segmenting the surface of the pelvis. Previous research showed that the impact of 8 mm of hip joint center mislocation could lead to errors in joint angles between 2$^{\circ }$ and 4$^{\circ }$^9^. These can be regarded as clinically relevant. Thus, the approximately 4 mm of error for reconstructing bone surface seem promising. However, it has to be clearly noted that our accuracy estimates are only based on the surface of the pelvis phantom and not on in-vivo measurements of the ASIS or the HJC position. Yet, our accuracy results are in good agreement with earlier research from Peters et al.^14^. They have validated a 3DFUS-based approach in localizing the HJC by using MRI derived HJC-distances and reported an accuracy of approximately 4 mm. However, this approach is limited as it only takes into account a distance derived from two systems as a surrogate outcome for accuracy. This approach does not allow to directly quantify the accuracy in reconstructing bone surface in 3D space. Our results extend their results and show that 3DFUS is able to reconstruct a surface within an accuracy of less than 4 mm. The accuracy estimates of our study are also in agreement with study results from the field of computer assisted orthopedic surgery. Dardenne et al.^24^ have evaluated the accuracy of an ultrasound-based device to measure patient-specific pelvic tilt on a testing mechanical bench. They reported an accuracy of approximately 1.1$^{\circ }$ (0.7) on average with a range of 0$^{\circ }$–4$^{\circ }$ . Similar to our phantom measurements Ottacher et al.^39^ have recently evaluated a 3DFUS based navigation system for inter-operative navigation. They performed intensive experiments and found that the 3DFUS-based system can localize 3D reconstructions of vertebral phantoms with an accuracy of approximately 2 mm. One reason why our results show slightly lower accuracy is, that our estimates also include errors of the manual identification of bony surface in each frame of the ultrasound images. The assessor who conducted the 3DFUS trials and digitized each frame was only moderately experienced. Thus, our results might still slightly underestimate the possible achievable accuracy as it can be difficult to accurately identify the bony surface in each ultrasound image. Nonetheless, our obtained results suggest that 3DFUS can serve as an accurate technique to register bony surface in individuals where palpation is difficult.

The identification of anatomical landmarks in motion capture data via 3DFUS might allow to fuse volumetric medical imagine data to gait analysis data. Gasparutto et al.^29^ have recently demonstrated that fusion of bi-planar X-ray to motion capture data significantly reduced the extrinsic variability due to skin marker misplacement. In their study, patients were equipped with the same pelvic markers during gait analysis and during a standing bi-planar X-ray. This allowed to fuse both data sets and re-calibrate the pelvic coordinate system during gait trials. 3DFUS could make the pelvic markers obsolete in the medical images. Segmented bone from a medical image (e.g. CT or MRI) could be easily fused to the position of the anatomical landmarks identified by 3DFUS in the motion capture data. Fusion could be performed by optimizing the point cloud of a volumetric medical image into the 3DFUS points by global point-wise optimization such as iterative closest point algorithms^40^. The combination of statistical shape models and sparse 3DFUS data of the pelvis might also allow to reconstruct the pelvic geometry^41,42^. Such approaches could open new perspectives for personalizing biomechanical models in 3D clinical gait analysis. However, applicability and feasibility of such approaches are subject to future research.

This study has some limitations which need to be recognized. So far, our 3DFUS-based approach seems to have image-quality related limitations. The utilized ultrasound probe showed a maximum penetration depth of approximately 6–7 cm. We experienced difficulties in obtaining good ultrasound images in six individuals, wherein we had to exclude 11 trials (legs) from the estimation of the HJC. The excluded cases had a BMI of $34.4 \pm 5.6$ kg/$\hbox {m}^2$. Reason for exclusion was that the femoral heads were located too deep below soft tissue and did not allow us to acquire images with sufficient quality. Therefore, technical robustness still needs optimization before such techniques can be implemented in clinical practice. The accuracy assessment was performed in distilled water with a plastic pelvis and therefore represented a “best case” scenario. Acoustic impedance might be different with soft tissue and bone. Further, our results only offer a rough estimate of the capability of 3DFUS in registering bony surface of the ASIS and HJC regions. Therefore, future studies should investigate the accuracy in a cadaver or in-vivo studies based on MRI or CT images. So far, the reconstructions of the ultrasound images were performed manually, frame-by-frame. This is a cumbersome and time-consuming task and is also prone to errors. In this study approximately 15–20 min were necessary to manually digitize the ultrasound images for one participant. Time is also necessary to record the 3DFUS trials, which adds additionally 5–10 min. In light of these time requirements, the possible gain in accuracy seems of rather low value in particularly time-sensitive settings such as clinical gait laboratories but should be of greater interest for research. Therefore, cutting down time expenditure is of great importance to further develop 3DFUS and make it feasible for clinical practice. Techniques in the research domains computer vision and machine learning might bear solutions to that problem by allowing the automatic or semi-automatic identification of bony landmarks in ultrasound images. First results based on computer vision techniques were recently presented by Jia et al.^19^. They have shown that 3DFUS bone segmentation can be performed to a certain extent with automated image processing techniques to reduce post-processing time. However, they did this for the trochanter major only, which is relatively easy to identify. Results presented in this study are limited to static calibration of a biomechanical model, but STA are especially an issue during movement trials. Therefore, 3DFUS-based approaches also need to be expanded to dynamic situations. First steps in that direction were already presented by Jia et al.^19^. Our study cohort only included children and adolescents. In several cases ossification of bone was still not completed and made the identification of the bone surface areas more difficult. Next to that, the relative size and composition of the pelvic bones in this young group might also limit generalizability to a certain extent.

In conclusion, our results give raise to the idea that an 3DFUS-based approach could serve as a reliable and accurate alternative in identifying anatomical landmarks such as the ASIS and the HJC in individuals especially with higher amounts of body fat. It also further corroborates the results of Peters et al.^15^ that 3DFUS can serve as an accurate alternative to identify the HJC position, especially when volumetric medical images such as bi-planar X-ray or CT scans, which expose patients to radiation, are not possible. Yet, 3DFUS is still time-consuming, needs a lot of manual post-processing, and is susceptible to outliers. Therefore, future developments should emphasis to increase technical robustness and further develop computer-assisted post-processing techniques to decrease processing time. This will help to make its application to clinical practice more viable.

## Supplementary Information

Supplementary Information 1.
