# Supplementary material for: 3D free-hand ultrasound to register anatomical landmarks at the pelvis and localize the hip joint center in lean and obese individuals
Source: Sci Rep. 2021 May 20;11:10650. doi: 10.1038/s41598-021-89763-7 (PMC8170673; doi:10.1038/s41598-021-89763-7)
Supplement: Supplementary file 1 — Supplementary Information 1. [file 41598_2021_89763_MOESM1_ESM.pdf]

Supplementary material: 3D free-hand ultrasound to register anatomical landmarks at the pelvis and localize the hip joint center in lean and obese individuals

Brian Horsak[1,*], Caterine Schwab[1], Sebastian Durstberger[2], Alexandra Thajer[3], Susanne Greber-Platzer[3], Hans Kainz[4], Ilse Jonkers[5], and Andreas Kranzl[2]

[1]St. Pölten University of Applied Sciences, Institute of Health Sciences, Austria
[2]Orthopedic Hospital Vienna-Speising, Laboratory of Gait and Movement Analysis, Austria
[3]Department of Pediatrics and Adolescent Medicine, Medical University of Vienna, Austria
[4]University of Vienna, Center for Sports Science and University Sports, Department of Biomechanics, Kinesiology and Applied Computer Science, Austria
[5]Human Movement Biomechanics Research Group, Department of Movement Sciences, KU Leuven, Belgium
[*]brian.horsak@fhstp.ac.at

ABSTRACT

3D free-hand ultrasound (3DFUS) is becoming increasingly popular to assist clinical gait analysis because it is cost- and time-efficient and does not expose participants to radiation. The aim of this study was to evaluate its reliability in localizing the anterior superior iliac spine (ASIS) at the pelvis and the hip joint centers (HJC). Additionally, we evaluated its accuracy to get a rough estimation of the potential to use of 3DFUS to segment bony surface. This could offer potential to register medical images to motion capture data in future. To evaluate reliability, a test-retest study was conducted in 16 lean and 19 obese individuals. The locations of the ASIS were determined by manual marker placement (MMP), an instrumented pointer technique (IPT), and with 3DFUS. The HJC location was also determined with 3DFUS. To quantify reliability, Intraclass Correlation Coefficients (ICCs), the Standard Error of Measurement (SEm), among other statistical parameters, were calculated for the identified locations between the test and retest. To assess accuracy, the surface of a human plastic pelvic phantom was segmented with 3DFUS in a distilled water bath in 27 trials and compared to a 3D laser scan of the pelvis. Regarding reliability, the MMP, but especially the IPT showed high reliability in lean (SEm: 2-3 mm) and reduced reliability in obese individuals (SEm: 6-15 mm). Compared to MMP and IPT, 3DFUS presented lower reliability in the lean group (SEm: 2-4 mm vs. 2-8 mm, respectively) but slightly better values in the obese group (SEm: 7-11 mm vs. 6-16 mm, respectively). Correlations between test-retest reliability and torso body fat mass (% of body mass) indicated a moderate to strong relationship for MMP and IPT but only a weak correlation for the 3DFUS approach. The water-bath experiments indicated an acceptable level of 3.5 (1.7) mm of accuracy for 3DFUS in segmenting bone surface. Despite some difficulties with single trials, our data give further rise to the idea that 3DFUS could serve as a promising tool in future to inform marker placement and hip joint center location, especially in groups with higher amount of body fat.

Supplementary material

The supplementary material includes the rain cloud and histogram plots for each 3DFUS bone segmentation trial (N = 27), and an illustration of each trial as overlay to the laser scan model, see Figures 1 and 2. The rain cloud and histogram plots allow the reader to inspect the variability in accuracy for each conducted segmentation trial. The overlays of the laser scan model with the 3DFUS segmentation give an overview of the scanned areas.

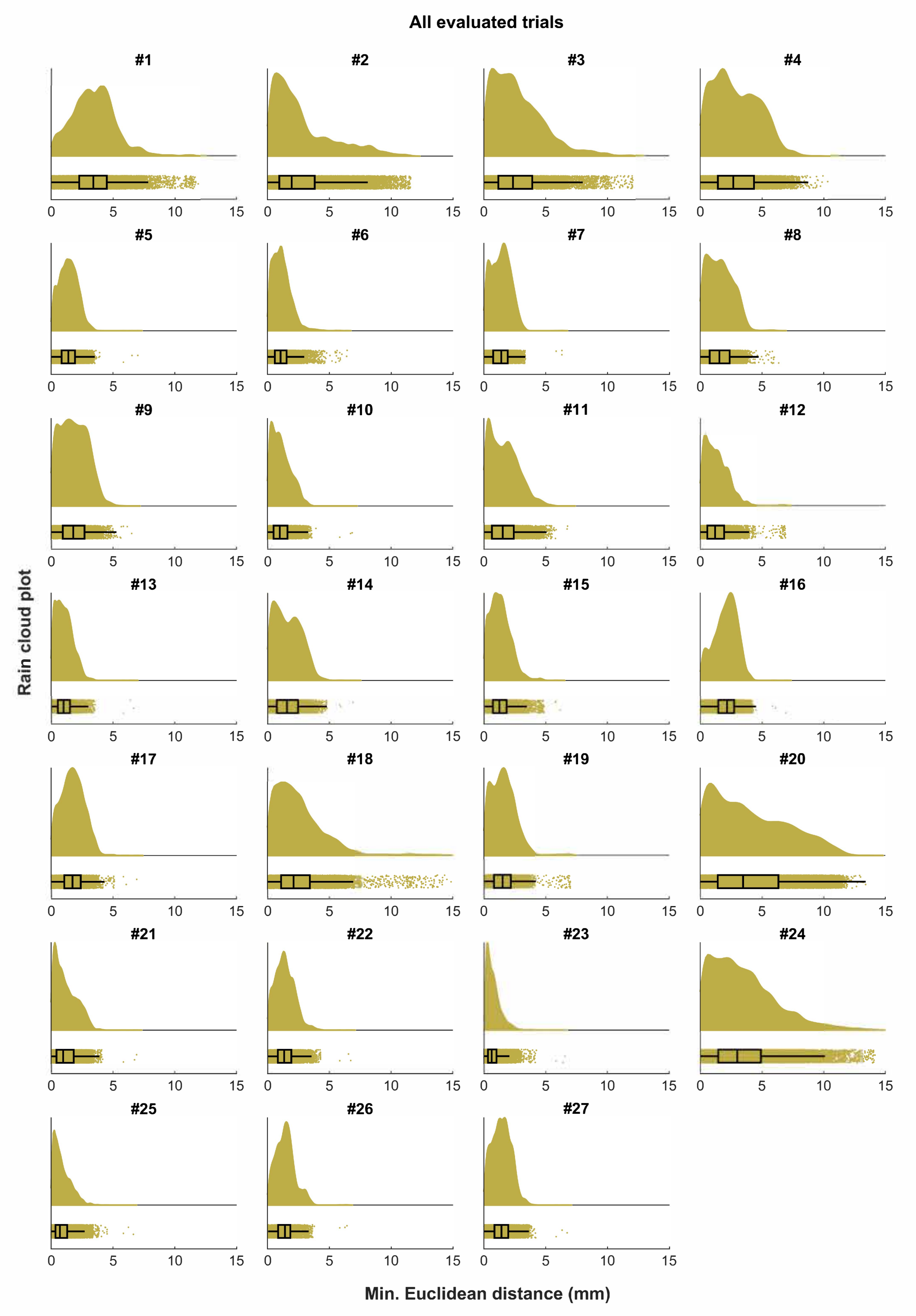

Figure 1. These plots show the minimum Euclidean distances, as rain cloud plots (left) and histograms (right), between each of the 27 3DFUS bone segmentation trials and the laser scan model (ground truth). The rain cloud plots combine an illustration of data distribution (probability density function), jittered raw data, and a boxplot (with the median).

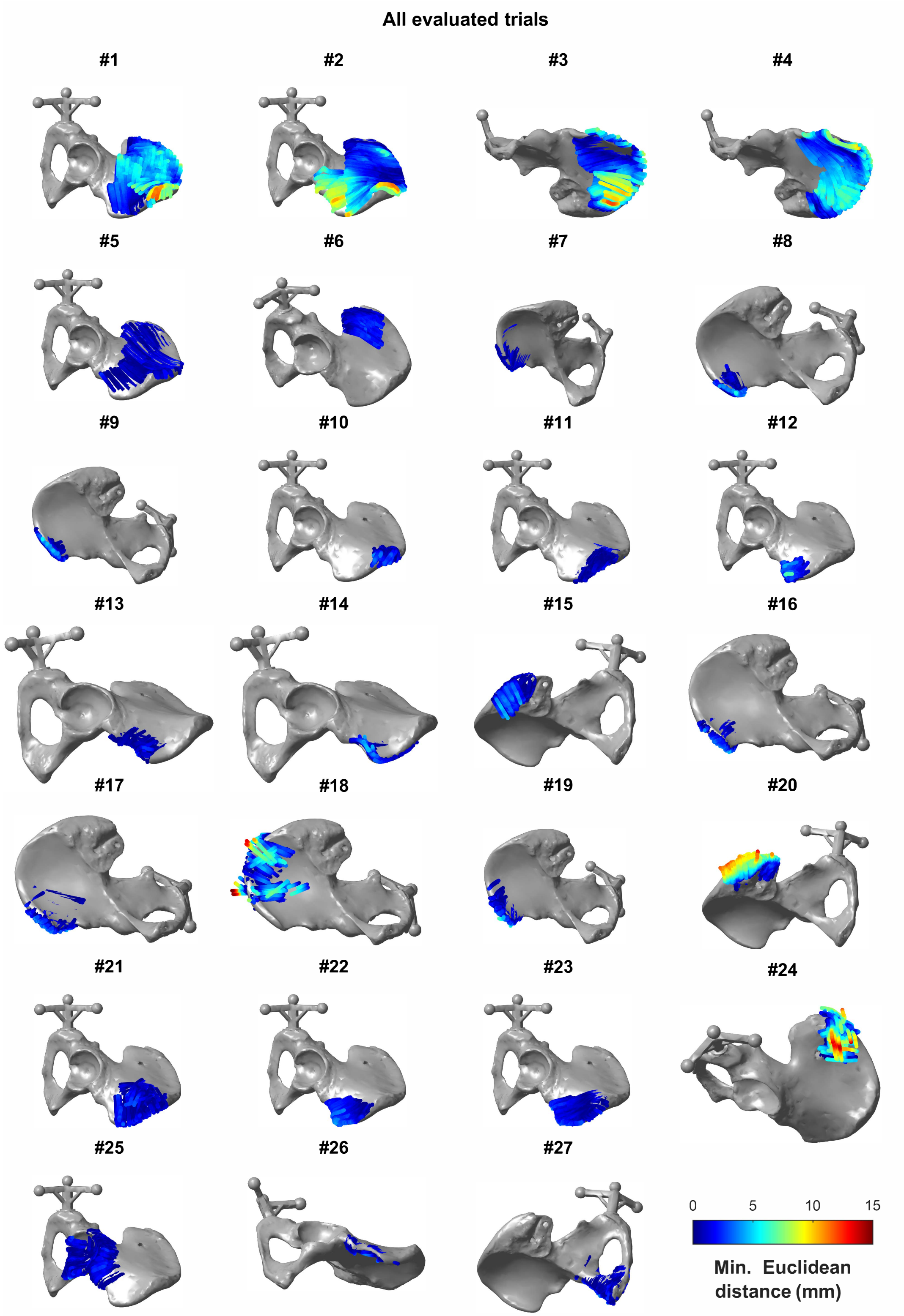

Figure 2. This supplementary figure shows the heatmap of the Euclidean distances between each of the 27 3DFUS trials and laser scan of the measurement phantom (ground truth). Red colors show greater differences and blue smaller ones.
